# Effect of Partial or Complete Replacement of Dietary Inorganic Trace Minerals Supplement with an Advanced Chelated Source on Nutrient Digestibility in Sheep

**DOI:** 10.3390/ani14223182

**Published:** 2024-11-06

**Authors:** Hossein Rajaei-Sharifabadi, Zahra Shokri, Mahdi Rohollahi, Mojtaba Yari, Saideh Fakharzadeh, Somayeh Kalanaky, Mohammad Hassan Nazaran, Gabriel de la Fuente Oliver, Ahmad Reza Seradj

**Affiliations:** 1 Department of Animal Science, Faculty of Agriculture, Malayer University, Malayer 6574184621, Iran; hrajaei@malayeru.ac.ir (H.R.-S.); zahrashokri.7596@gmail.com (Z.S.); mehdiroholahi33@gmail.com (M.R.); mojyari@gmail.com (M.Y.); 2 Departament de Ciència Animal, Universitat de Lleida-Agrotecnio-CERCA Center, 25198 Lleida, Spain; reza.seradj@udl.cat; 3 Department of Research and Development, Sodour Ahrar Shargh Co., Tehran 1415944341, Iran; sfakharzade@nanochelatingtechnology.com (S.F.); skalanaky@nanochelatingtechnology.com (S.K.)

**Keywords:** advanced chelate compounds technology, in situ rumen degradability, mineral solubility, nutrient digestibility, trace minerals

## Abstract

The delicate balance of trace mineral supplementation is critical for optimizing rumen function and overall ruminant health. This study evaluated the solubility of an advanced chelate technology-based supplement and assessed its impact on rumen degradability and apparent total tract digestibility (ATTD) when replacing inorganic sources. The solubility of the advanced trace minerals supplement (ACTM) was assessed at pH 5 and pH 2. In situ ruminal degradability of dry matter (DM), organic matter (OM), and fiber fractions was evaluated using two fistulated rams fed diets supplemented with either ACTM or inorganic trace minerals. ATTD was determined in 6 lambs fed diets supplemented with 100% ACTM, 50% ACTM, and 50% inorganic (50% ACTM), or 100% inorganic sources in a Latin square design. Results showed solubilities ranging from 6.75% to 11.81% at pH 5, increasing to 69.24% to 80.47% at pH 2. ACTM supplementation significantly enhanced the rumen degradability of neutral detergent fiber (NDF) and acid detergent fiber (ADF) at 6 h of incubation (*p* ≤ 0.05). The 100% ACTM treatment significantly decreased rumen pH (*p* = 0.051) and improved DM, OM, NDF, and ADF digestibility, as well as trace mineral absorbability compared to 100% inorganic (*p* ≤ 0.05). These findings highlight the potential of ACTM supplementation to enhance ruminal degradability, promote better trace mineral absorption, and improve the ATTD of nutrients compared to inorganic sources.

## Simple Summary

Improving trace mineral supplementation in ruminants is crucial for optimal health and performance, although traditional inorganic sources may have some drawbacks, particularly regarding rumen function. We investigated an advanced chelate technology-based trace mineral (ACTM) supplement containing 7 trace minerals (Bonza^®^plex 7). In the in vitro assay, ACTM presented low solubility at rumen pH but high solubility at abomasal pH, suggesting an increase in the rumen bypass and minimal negative effects on ruminal fermentation. In situ experiments showed that ACTM enhanced early fiber degradability in the rumen. When fed to lambs, ACTM improved nutrient digestibility and mineral absorption compared to inorganic sources. However, partial replacement of inorganic minerals with ACTM showed unconclusive results. These findings suggest that ACTM supplementation can enhance rumen function and mineral utilization in ruminants, potentially leading to improved animal performance. Further research is warranted to determine optimal inclusion rates and elucidate the effects of ACTM supplementation on rumen fermentation parameters, microbial population dynamics, and long-term animal performance across various ruminant production systems.

## 1. Introduction

Trace minerals, such as Cu, Zn, Mn, and Co, play crucial roles in ruminant nutrition, particularly in supporting optimal rumen function and overall animal health and productivity. These minerals are essential for various enzymatic processes and serve as cofactors in numerous metabolic pathways critical for both the host animal and ruminal microorganisms [1,2]. However, the complex interplay between trace minerals supplementation and rumen function [3,4] presents a significant challenge in ruminant nutrition. Although rumen microorganisms require these minerals (e.g., Zn, Cu, Co), their demand is substantially lower than the overall nutritional needs of the host animal [1]. High concentrations of trace minerals have been shown to negatively impact ruminal fermentation, reducing fiber digestibility and altering volatile fatty acid ratios [5,6]. Conversely, deficiencies in certain micronutrients can lead to reduced cellulose digestion and impaired rumen function [7]. Thus, it is required a delicate balance in trace minerals supplementation to avoid adverse effects on rumen microbial activity and overall animal health.

The source and solubility of trace minerals in the rumen environment significantly influence their availability to microorganisms and their impact on fermentation processes. Traditional inorganic sources, such as sulfates and oxides, differ in their solubility and bioavailability compared to organic sources complex with amino acids or proteins and newer forms like hydroxy minerals. Inorganic sources generally have high solubility but lower absorption rates compared to organic sources. Hydroxy minerals, on the other hand, have low solubility in the pH conditions of the rumen but become more soluble in the acidic environment of the abomasum [8,9,10]. Recent research has highlighted the potential benefits of using less soluble forms of trace minerals, such as hydroxy forms, which have demonstrated improved fiber digestibility and favorable alterations in volatile fatty acid production patterns [4,11,12,13]. These findings indicate that the strategic selection of trace mineral sources with both low ruminal and high intestinal solubility could optimize rumen fermentation and nutrient utilization in high-producing ruminants [14]. This approach may open new avenues for enhancing rumen function and overall animal performance through targeted trace mineral supplementation. Furthermore, improved trace mineral absorption can potentially reduce environmental pollution by decreasing mineral excretion. Enhanced bioavailability of trace minerals leads to more efficient utilization by the animal, resulting in lower concentrations of these elements in manure and other waste products.

Advanced chelate compounds technology [15] represents a significant innovation in trace mineral supplementation for livestock nutrition. This self-assembly method involves the polymerization of organic acids under controlled conditions, allowing mineral elements to bond with specific coordination sites based on their affinity [16]. Unlike traditionally available organic mineral sources that use amino acids or peptides as ligands, this technology uniquely employs organic acids as the chelating agents. This distinction is crucial, as it influences the properties and behavior of the resulting mineral complexes. Organic acids can traverse the rumen epithelium via simple diffusion, a process dependent on the concentration gradient between rumen contents and the bloodstream. Absorption efficiency is higher for acids in their non-ionized (protonated) form, with rumen pH playing a crucial role; lower pH favors the more absorbable (i.e., non-ionized) forms [17]. The interaction between organic acids and metal elements significantly impacts absorption and bioavailability. When organic acids bind metals, they form chelates, which protect the metals from creating insoluble compounds. This chelation enhances metal transport across the intestinal lining and often improves their bioavailability, allowing for better utilization by the body post-absorption. The improved bioavailability is attributed to the chelated form being more readily recognized and taken up by specific transporters in the gut [17]. Previous studies indicated that trace mineral supplements manufactured based on this technology are stable at pH 4 and pH 9 [18]. These properties may offer some benefits for ruminants in terms of lower negative impacts on rumen fermentation.

The advanced chelated trace minerals (ACTM) supplements have shown promising results in studies on poultry, improving growth performance, bone health, and egg quality [18,19]. However, the potential benefits of ACTM extend beyond avian species, with growing interest in their application for ruminant nutrition. Emerging evidence suggests that ACTM may have the potential to improve health and productivity in ruminants. Studies by Dehghan-Banadaky et al. [20] and Mousavi-Haghshenas et al. [21] have reported positive effects on various aspects of ruminant performance and metabolism.

Despite these promising characteristics, there is a notable gap in research regarding the behavior of ACTM in the rumen environment. The unique physiological conditions of the rumen, including its pH fluctuations and diverse microbial population, compel a thorough evaluation of the solubility and stability of this form of trace minerals. Understanding how these factors affect the release and availability of minerals is crucial for optimizing their use in ruminant nutrition without compromising rumen fermentation processes. Therefore, the present study aimed to evaluate the solubility of a dietary ACTM supplement designed for ruminants (Bonza^®^plex 7) at two physiologically relevant pH levels, as well as to determine its effects on rumen degradability and total trace digestibility of nutrients when partially or completely replaced with inorganic sources.

## 2. Materials and Methods

### 2.1. Experiment I: In Vitro Solubility Assessment of Advanced Chelated Trace Minerals Supplement

The solubility of trace minerals in the ACTM supplement (Bonza^®^plex 7, Sodour Ahrar Shargh Company, Tehran, Iran) was evaluated in vitro at two pH levels to simulate different physiological conditions: pH 5 (approximating ruminal conditions) and pH 2 (approximating abomasal conditions). The procedure was adapted from methods described by Brown and Zeringue [8] and Cao et al. [9]. Briefly, KH_2_PO_4_ buffer (pH 5) was prepared using 0.1 M KH_2_PO_4_, adjusted to pH 5 with 1 M NaOH, while HCl-KCl buffer (pH 2) was prepared by mixing 0.2 M KCl and 0.2 M HCl. For each pH condition, 50 mL of buffer solution containing 0.5 mg/mL Bonza^®^plex 7 was prepared in triplicate. The solutions were incubated in a water bath at 39 °C (to mimic body temperature) for 24 h. After incubation, the solutions were immediately filtered through Whatman No. 42 filter paper using a vacuum filtration system. The filter was washed with 25 mL of deionized water to ensure complete recovery of soluble minerals. The filtrate was collected in acid-washed volumetric flasks and diluted to a final volume of 100 mL with deionized water and stored at 4 °C for further analysis.

### 2.2. Experiment II: In Situ Ruminal Degradability of Nutrients

The in situ experiment was conducted at the research farm of the Department of Animal Sciences, Malayer University. Two ruminally fistulated Afshari rams were used in a 2 × 2 Latin square design (two experimental treatments and two periods). The rams used in this experiment had been fistulated approximately 2 years prior to the study’s commencement, ensuring full recovery and adaptation to their fistulas before experimental procedures began. All fistulation procedures were carried out under strict veterinary supervision with comprehensive post-surgical care, including regular monitoring of the surgical site, appropriate pain management, and infection prevention measures until complete healing was achieved. Throughout the study, the animals received consistent veterinary oversight to ensure their health and well-being. The rams were housed in individual pens (2 m × 1 m). A basal diet containing 30% forage and 70% concentrate (Table 1) was formulated according to NRC [22] recommendations and offered to the animals twice daily at 0800 and 1600 h, at a rate of 3% of body weight.

The basal diet was supplemented with equal amounts of trace minerals from either an ACTM source (ORG; 1 g/kg DM Bonza^®^plex 7, Sodour Ahrar Shargh Co., Tehran, Iran) or inorganic sources (IOR). Both supplements provided 51 mg Zn, 28 mg Mn, 18 mg Cu, 8 mg Fe, 1.9 mg Co, 0.5 mg Cr, and 0.3 mg Se per kg DM of the basal diet.

Following 10 days of acclimation, each experimental period consisted of 13 days for adaptation (washout), followed by 2 days for the in situ procedure. The washout period allowed for the reset of rumen microbiota and adaptation of the rumen environment to the new dietary treatment, thus minimizing potential carryover effects between experimental phases. Feed samples were ground through a 2 mm sieve using a hammer mill. Nylon bags (5 × 10 cm) were filled with 2 g of ground feed sample. For each incubation time point, four bags were prepared (one empty bag as a blank and three bags containing a feed sample). The bags were simultaneously inserted into the rumen 1 h after morning feeding and removed at 6, 12, 24, and 36 h post-incubation. Upon removal, bags were immediately immersed in ice-cold water (4 °C) to halt microbial activity and transported to the laboratory on ice. In the laboratory, bags were rinsed with tap water until the effluent ran clear, followed by two rinses with distilled water. Three additional bags containing feed samples were also prepared but not inserted into the rumen. These bags were washed using the same procedure as the incubated bags to estimate the immediately soluble fraction. The washed bags were then dried in a forced-air oven at 70 °C for 48 h. Dried residues were weighed and stored at −20 °C for further analyses.

### 2.3. Experiment III: Apparent Total Tract Digestibility of Nutrients

This experiment was conducted at the research farm of the Department of Animal Sciences, Malayer University. Six Afshari ram lambs with an average initial body weight of 40 ± 2.6 kg were used in a 3 × 3 Latin square design with three experimental treatments and three periods. The animals were housed in individual metabolic cages (50 cm × 100 cm) constructed of steel and equipped with plastic water troughs and metal feed troughs. A plastic net was installed beneath each cage to facilitate fecal collection.

Prior to the experiment, lambs were vaccinated against enterotoxemia and foot-and-mouth disease. Animals were acclimated to the experimental conditions and diet for 10 days before the start of the trial. Each experimental period consisted of 10 days for adaptation (washout), followed by 5 days for sampling. A basal diet containing 30% forage and 70% concentrate (Table 1) was formulated according to NRC [22] recommendations. The diet was offered twice daily at 0800 and 1600 h, at a rate of 3% of body weight. Fresh drinking water was available ad libitum throughout the experiment.

Three treatments were evaluated in this experiment. The first treatment (100% OR) consisted of trace minerals supplied entirely from an advanced chelated source, with each animal receiving 1 g Bonza^®^plex/kg DM. The second treatment (50% OR) provided trace minerals at an equal total amount of the 100% OR treatment but from a combination of advanced chelated and inorganic sources, where half of the trace minerals (0.5 g Bonza^®^plex7/kg DM) came from the chelated source and the other half from inorganic sources. The third treatment (100% IOR) supplied trace minerals exclusively from inorganic sources, matching the total trace mineral content provided in the other treatments. Trace mineral supplements were mixed daily with the concentrate portion of each lamb’s ration before feeding.

The supplementation of 1 g Bonza^®^plex 7 provided 51 mg Zn, 28 mg Mn, 18 mg Cu, 8 mg Fe, 1.9 mg Co, 0.5 mg Cr, and 0.3 mg Se per kg DM of the diet. For the IOR treatment, equivalent amounts of trace minerals were supplied using inorganic sources: 159 mg zinc sulfate (32% Zn), 82 mg manganese sulfate (34% Mn), 90 mg copper sulfate (20% Cu), 42 mg iron sulfate (19% Fe), 9 mg cobalt sulfate (19.5% Co), 2.5 mg chromium chloride (19.5% Cr), and 1 mg sodium selenite (42% Se).

On day 11 of each experimental period, rumen fluid samples were collected 2 h after morning feeding using a gastric tube connected to a vacuum pump. The initial 100 mL of fluid was discarded to minimize saliva contamination. Approximately 200 mL of rumen fluid was then collected, and pH was measured immediately using a portable pH meter.

To determine apparent total tract nutrient digestibility (ATTD), total fecal output was collected daily for four consecutive days (days 12 to 15 of each period). After weighing, a 200 g subsample was taken daily, dried at 60 °C for 48 h in a forced-air oven, and stored at −20 °C for further analysis. Feed samples were also collected on days 12 to 15 of each period, dried similarly, and stored at −20 °C pending analysis.

### 2.4. Chemical Analyses

All feed and fecal samples from Experiment II and Experiment III were ground using a hammer mill equipped with a 2 mm sieve and subjected to chemical analysis using standard methods [23]. DM was determined by oven-drying at 105 °C for 24 h, and organic matter (OM) was calculated as the difference between DM and ash content after incineration at 600 °C for 6 h. Neutral detergent fiber (NDF) and acid detergent fiber (ADF) were analyzed, according to Van Soest et al. [24], without α-amylase and expressed inclusive of residual ash. Trace mineral concentrations were determined using Inductively Coupled Plasma Optical Emission Spectrometry (ICP-OES). The instrument was calibrated using multi-element standard solutions prepared from certified single-element standards. The following wavelengths were used for element quantification: Zn (213.856 nm), Cu (324.754 nm), Mn (257.610 nm), Fe (259.940 nm), Co (228.616 nm), Cr (267.716 nm), and Se (196.090 nm).

### 2.5. Statistical Analysis

The normality of the data distribution was assessed using the PROC UNIVARIATE procedure of SAS [25] (SAS 9.1, SAS Institute Inc., Cary, NC, USA). Analysis of variance (ANOVA) was conducted using the MIXED procedure of SAS, employing a 2 × 2 Latin square design for Experiment II and a 3 × 3 Latin square design for Experiment III. Least squares means were compared using the pdiff option at a significance level of *p* ≤ 0.05. Effects were considered statistically significant at *p* ≤ 0.05, while trends were noted for 0.05 < *p* ≤ 0.10.

## 3. Results

### 3.1. Trace Minerals Solubility

Table 2 presents the solubility of trace minerals in the ACTM supplement at both pH 5 and pH 2. Solubility values for Fe and Cr were excluded from further analysis due to possible contamination or analytical discrepancies arising from the preparation of buffers or mineral supplements. At pH 5, the solubilities ranged from 6.75% for Co to 11.81% for ZN. However, solubility increased substantially at pH 2, with values ranging from 69.24% (for Co) to 80.47% (for Mn).

### 3.2. In Situ Rumen Degradability

**Figure 1:**
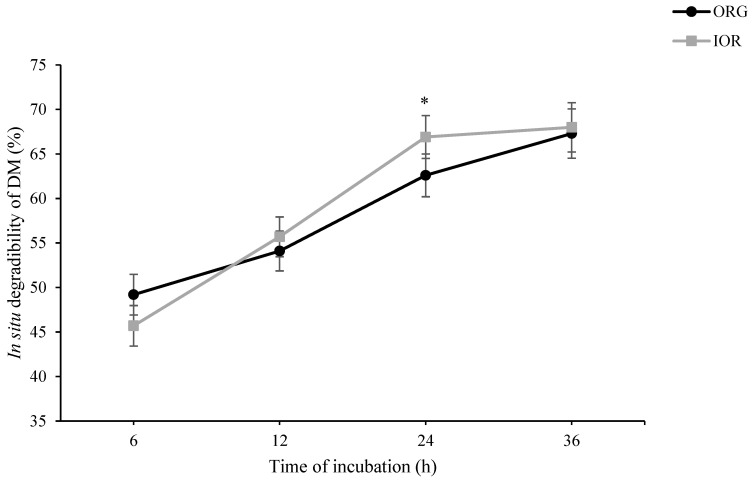
Effect of advanced chelated trace minerals supplements on in situ rumen degradability of dry matter (DM). ORG: Diet supplemented with trace minerals entirely from an advanced chelated source (1 g/kg DM Bonza^®^plex 7); IOR: Diet supplemented with trace minerals entirely from inorganic sources. * Indicates a significant difference between treatments at *p* ≤ 0.05.

**Figure 2:**
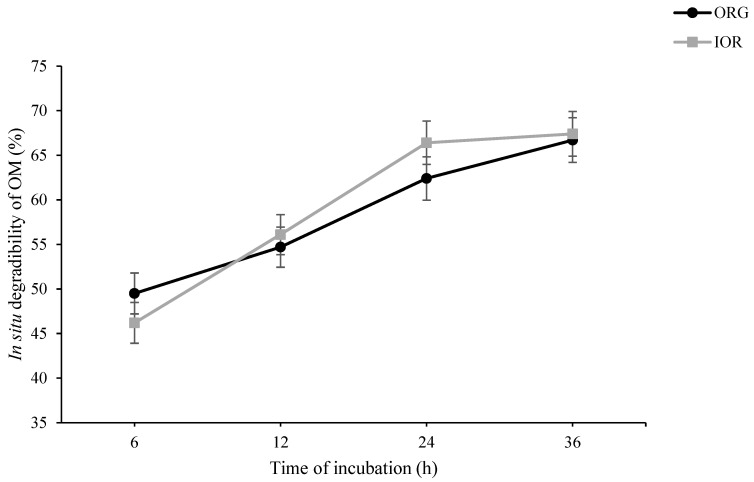
Effect of advanced chelated trace minerals supplements on in situ rumen degradability of organic matter (OM). ORG: Diet supplemented with trace minerals entirely from an advanced chelated source (1 g/kg DM Bonza^®^plex 7); IOR: Diet supplemented with trace minerals entirely from inorganic sources.

**Figure 3:**
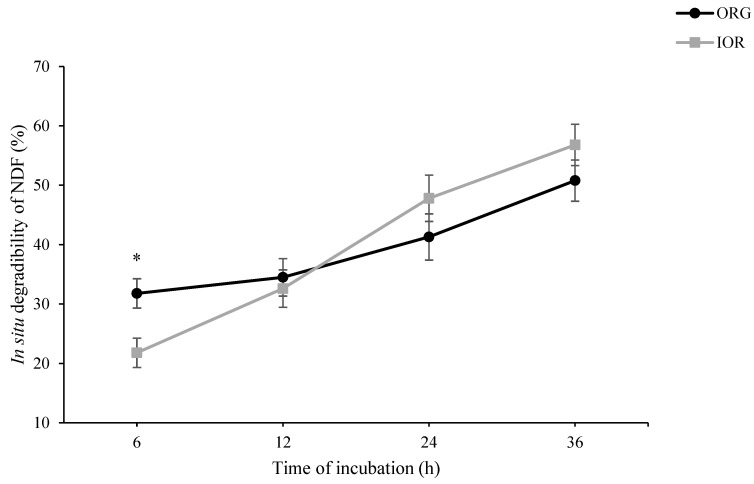
Effect of advanced chelated trace minerals supplements on in situ rumen degradability of neutral detergent fiber (NDF). ORG: Diet supplemented with trace minerals entirely from an advanced chelated source (1 g/kg DM Bonza^®^plex 7); IOR: Diet supplemented with trace minerals entirely from inorganic sources. * Indicates a significant difference between treatments at *p* ≤ 0.05.

**Figure 4:**
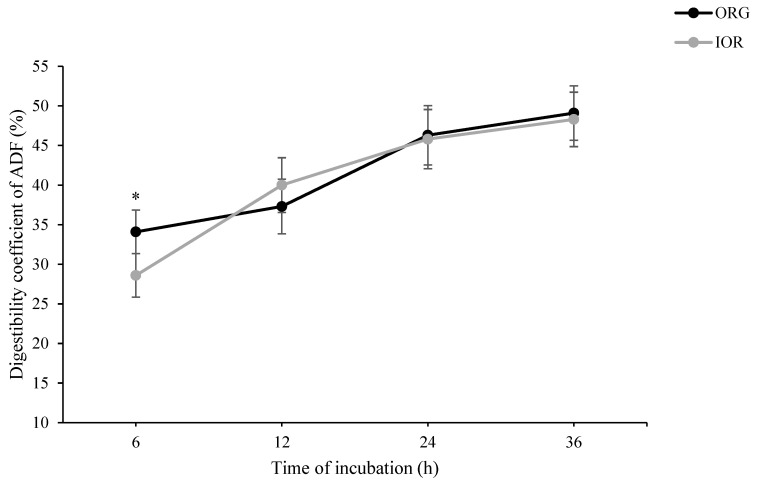
Effect of advanced chelated trace minerals supplements on in situ rumen degradability of acid detergent fiber (ADF). ORG: Diet supplemented with trace minerals entirely from an advanced chelated source (1 g/kg DM Bonza^®^plex 7); IOR: Diet supplemented with trace minerals entirely from inorganic sources. * Indicates a significant difference between treatments at *p* ≤ 0.05.

As shown in Figure 1, in situ rumen degradability of DM at h 6 of incubation tended to be higher in ORG than in IOR treatment (*p* = 0.087). At h 24 of incubation, however, a tendency toward higher in situ rumen degradability of DM was observed in IOR compared to ORG (*p* = 0.054). A similar pattern was also observed for in situ rumen degradability of OM (Figure 2). Regarding both NDF (Figure 3) and ADF (Figure 4), ORG treatment showed significantly higher in situ rumen degradability compared to IOR at h 6 of incubation. No significant differences were detected between ORG and IOR for in situ rumen degradability of NDF and ADF at h 12, 24, and 36 of the incubation.

### 3.3. Apparent Total Trace Digestibility

Table 3 shows the rumen pH and ATTD of nutrients in Experiment III. Ruminal pH was significantly lower in 100% OR treatment compared to 100% IOR. No significant differences were observed in 50% OR and both 100% OR and 100% IOR treatments for ruminal pH. Replacing completely inorganic trace mineral sources with the advanced chelated trace mineral source improved ATTD of DM, OM, NDF, and ADF. Although ATTD of nutrients in 50% OR was numerically higher than 100% IOR; however, differences were non-significant between these two treatments. Absorbability values of Cu and Fe were significantly higher in 100% OR than both 50% OR and 100% IOR (Table 4). The highest absorbability values of Zn and Mn were observed in 100% OR, followed by 50% OR and 100% IOR, with significant differences between 100% OR and 100% IOR. There was a significant difference between 100% IOR and both 100% OR and 50% OR for the absorbability of Se. Absorbability data for Cr was excluded from the analysis due to suspected contamination or inconsistencies during sample preparation, which could have affected the accuracy of these measurements.

## 4. Discussion

The solubility of trace minerals in the ACTM supplement demonstrated a distinct pH-dependent pattern, with significant implications for ruminant nutrition. At pH 5, approximating rumen conditions, mineral solubility was low (6.75% to 11.81%), while it increased substantially at pH 2, simulating the abomasal environment (69.24% to 80.47%). This pH-dependent profile aligns with the behavior of hydroxy forms of trace minerals, as reported in previous studies. Cao et al. [9] observed lower water solubility for Zn hydroxy chloride compared to ZnSO4, while Spears et al. [13] found that Cu from Cu hydroxy chloride was relatively insoluble (0.6%) at neutral pH but highly soluble (81.4%) at low pH. In vivo studies have further corroborated these findings. Caldera et al. [14] reported higher Cu concentration in rumen fluid supernatant with CuSO4 compared to Cu hydroxy, indicating lower ruminal solubility of Cu hydroxy. Similarly, Genther and Hansen [10] observed higher ruminal solubility of CuSO4 compared to Cu hydroxy. For Zn sources, Caldera et al. [14] found lower ruminal solubility for Zn hydroxy compared to ZnSO4, although Genther and Hansen [10] reported contrasting results at higher supplementation levels.

The low solubility of ACTM at rumen pH is advantageous, as it suggests these minerals would remain largely undissociated in the rumen environment, potentially reducing interference with microbial processes and the formation of insoluble complexes with other dietary components. This characteristic is crucial for minimizing negative impacts on microbial activity and overall rumen function. Conversely, the increased solubility at pH 2 indicates enhanced availability for absorption in the abomasum and proximal duodenum, potentially improving overall mineral bioavailability. This pH-dependent solubility profile suggests a targeted release mechanism rather than a true bypass. The minerals become more soluble and thus more bioavailable as they move through the digestive tract, potentially improving overall mineral absorption and utilization. However, it is important to note that while these in vitro solubility results are promising, they do not directly translate to in vivo bioavailability. Clarkson et al. [26] emphasized the significant effect of the rumen environment on the solubility of different trace mineral sources. Therefore, further in vivo studies are necessary to confirm these hypotheses and evaluate the actual bioavailability and utilization of these minerals in ruminants, considering the complex interactions within the digestive system.

The in situ rumen degradability results provide further insight into the rumen-protective nature of the ACTM supplement (ORG treatment). The tendency for higher DM and OM degradability in the ORG treatment at 6 h, aligned with significantly higher NDF and ADF degradability, suggests that trace minerals from the ACTM source may initially be more accessible to rumen microbes or, more likely, had no negative effect on microbial activity in the rumen, particularly fibrolytic bacteria. However, the lower DM and OM degradability at 24 h of incubation in the ORG compared to the IOR treatment may be associated with the lower remaining degradable substrate in the ORG treatment, suggesting more efficient early degradation. These results align with the lower rumen pH observed in the 100% OR treatment in Experiment III (Table 3), indicating a higher fermentation capacity for the diet supplemented with ACTM sources compared to inorganic sources. It is important to note that while the pH was lower in the ACTM treatment, it remained within the safe range for maintaining rumen health (pH ≥ 5.5), mitigating concerns about potential sub-acute ruminal acidosis. Collectively, these findings suggest that replacing inorganic sources of trace minerals with the ACTM source has the potential to enhance rumen degradability and fermentation efficiency. This improvement in ruminal function could lead to better nutrient utilization and potentially improved animal performance.

Research on the effects of trace mineral sources on rumen microbial communities, particularly hydroxy sources, is still limited and has yielded varied findings [4]. For instance, Zn supplementation has shown varying impacts depending on its source. Ishaq et al. [27] reported that Zn amino acid complexes reduced bacterial diversity while ZnSO_4_ had no significant effect. In lambs, Zn amino acid supplementation decreased total bacterial populations but increased specific microbial populations such as *Ruminococcus albus* and *Streptococcus bovis* without affecting methanogenic *Archaea* [28]. Conversely, in dairy cows, increasing levels of coated ZnSO_4_ enhanced total bacterial populations and fibrolytic bacteria, including *Ruminococcus albus* and *Fibrobacter succinogenes* [29]. Cu supplementation, in the form of CuSO_4_, has been shown to increase fibrolytic bacteria and enzyme activity in dairy cows while reducing *Prevotella ruminicola* populations [30]. Mn supplementation also affected microbial diversity and enzymatic activity, with organic Mn reducing microbial diversity in ewe lambs, while inorganic Mn had no significant impact [31].

However, it is important to note that in some studies, such as those on beef heifers and heat-stressed dairy steers, trace mineral supplementation did not notably alter microbial diversity or methanogenic populations [4]. These contrasting findings underscore the complexity of trace minerals’ effects on rumen microbiota, which appear to vary depending on the mineral form, dosage, and animal species. Although this in situ study demonstrated the positive effects of ACTM on rumen degradability, further research is needed to fully elucidate its impact on microbial activity. Future studies should specifically investigate how ACTM influences microbial diversity, population dynamics, and activity in the rumen across different ruminant species and production types (e.g., dairy cattle, beef cattle, sheep, and goats). Additionally, it would be valuable to explore the effects of ACTM under varying dietary compositions and environmental conditions, such as different forage-to-concentrate ratios, feed quality, and heat stress scenarios. Such comprehensive studies would enhance our understanding of how ACTM affects microbial ecosystems within the rumen and how these interactions subsequently influence nutrient utilization, animal performance, and health.

In the context of our study, the improved early degradability observed with the ACTM supplement aligns with some of these previous findings, particularly those showing enhanced fibrolytic bacterial activity with certain forms of trace mineral supplementation. However, the mechanisms behind these effects and their long-term implications for rumen function and overall animal performance require further investigation. Future studies should aim to elucidate the specific interactions between ACTM and rumen microbiota, potentially employing advanced molecular techniques to provide a more comprehensive understanding of these complex relationships.

The results of the ATTD trial provide more evidence for the positive impact of the ACTM supplement on nutrient digestibility. The complete replacement of inorganic minerals with ACTM (100%OR treatment) led to improved ATTD of DM, OM, NDF, and ADF. These results are in line with the results of the in situ trial, where rumen degradability of nutrients was higher in ORG than in IOR treatment. Furthermore, the significantly higher absorbability values for Cu, Fe, Zn, Mn, and Se in the 100%OR treatment compared to the 100%IOR treatment further confirm the superior bioavailability of the chelated minerals. These findings suggest that the ACTM not only enhances mineral availability but may also positively influence overall nutrient utilization, possibly through improved ruminal fermentation or post-ruminal digestive processes. There is some evidence that supplementing ruminants with organic trace minerals improves the activity of digestive enzymes. For instance, Zhang [32] reported that supplementing sheep diets with oligosaccharide chelates of trace minerals at a rate of 0.1% increased the activity of digestive enzymes such as amylase, lipase, and trypsin. It is possible that in this research, chelated elements increased the activity of digestive enzymes and, as a result, increased the digestibility of dry matter and organic matter.

## 5. Conclusions

In conclusion, our findings demonstrate that the ACTM supplement exhibited low solubility at ruminal pH and high solubility in more acidic conditions, suggesting a potential rumen-protective effect while enhancing post-ruminal absorption. Partial or complete replacement of inorganic trace mineral sources with ACTM improved rumen degradability and total tract digestibility of nutrients, as well as increased mineral absorbability. These results collectively suggest that the ACTM supplement offers a promising approach to trace mineral nutrition in ruminants. However, further research is warranted to evaluate long-term effects and performance outcomes across various ruminant species and production systems, as well as to assess the impact of ACTM supplementation on rumen microbiota composition and diversity, which could provide valuable insights into the mechanisms underlying the observed improvements in nutrient utilization.

## Figures and Tables

**Table 1:** Ingredients and chemical composition of the basal diet (dry matter basis, %).

Ingredient	Amount
Alfalfa hay	20
Wheat straw	10
Barley grain	33
Corn grain	10
Soybean meal	10
Wheat bran	15
Calcium carbonate	0.7
Sodium bicarbonate	0.7
Salt	0.3
Vitamin premix	0.3
Chemical composition	
Dry matter	96.67
Organic matter	92.40
Crud protein	13.91
Neutral detergent fiber	30.3
Acid detergent fiber	18.6

**Table 2:** Solubility of minerals in advanced chelated trace minerals supplements at different pH.

Mineral	Concentration in Medium, mg/mL	Solubility, %	
		pH 5	pH 2
Cu	0.288	8.79 (±1.08)	70.31 (±8.62)
Zn	0.816	11.81 (±0.97)	78.90 (±6.33)
Mn	0.448	11.67 (±1.70)	80.47 (±11.71)
Co	0.027	6.75 (±0.83)	69.24 (±8.49)
Se	0.005	9.14 (±0.92)	77.78 (±7.82)

**Table 3:** Effect of advanced chelated trace minerals supplement on rumen pH and apparent total tract digestibility (ATTD) of nutrient.

Item	Treatment ^1^			SEM ^2^	*p* Value
	100% OR	50% OR	100% IOR		
Rumen pH	6.07 ^b^	6.14 ^ab^	6.33 ^a^	0.11	0.051
ATTD, %					
Dry matter	69.0 ^a^	65.3 ^ab^	63.9 ^b^	1.92	0.047
Organic matter	68.3 ^a^	63.3 ^b^	61.1 ^b^	2.01	0.019
Neutral detergent fiber	61.7 ^a^	55.4 ^b^	56.2 ^b^	1.77	0.016
Acid detergent fiber	46.9 ^a^	35.2 ^b^	31.0 ^b^	1.69	0.028

^1^ 100% OR: Trace minerals entirely from advanced chelated source (1 g/kg DM Bonza^®^plex 7); 50% OR: Trace minerals from a combination of advanced chelated (0.5 g/kg DM Bonza^®^plex 7) and inorganic sources; 100% IOR: Trace minerals entirely from inorganic sources, matching total trace mineral content of other treatments. ^2^ SEM: standard error of the mean. ^a,b^ Values with different superscripts within a row are significantly different (*p* ≤ 0.05).

**Table 4:** Effect of advanced chelated trace minerals supplements on total tract absorbability of trace minerals.

Mineral	Treatment ^1^			SEM ^2^	*p* Value
	100% OR	50% OR	100% IOR		
Cu	24.61 ^a^	17.67 ^b^	15.67 ^b^	2.49	<0.01
Zn	61.36 ^a^	56.69 ^ab^	51.16 ^b^	3.10	<0.01
Mn	32.20 ^a^	27.29 ^ab^	24.39 ^b^	2.32	<0.01
Fe	36.20 ^a^	25.88 ^b^	24.37 ^b^	2.74	<0.01
Co	61.71	60.99	60.31	3.14	0.793
Se	29.40 ^a^	28.05 ^a^	21.46 ^b^	2.47	<0.01

^1^ 100% OR: Trace minerals entirely from advanced chelated source (1 g/kg Bonza^®^plex 7); 50% OR: Trace minerals from a combination of advanced chelated (0.5 g/kg Bonza^®^plex 7) and inorganic sources; 100% IOR: Trace minerals entirely from inorganic sources, matching total trace mineral content of other treatments. ^2^ SEM: standard error of the mean. ^a,b^ Values with different superscripts within a row are significantly different (*p* ≤ 0.05).

## Data Availability

The raw data supporting the conclusions of this article will be made available by the authors upon request.
